# Visualising key information and communication technologies (ICT) indicators for children and young individuals in Europe

**DOI:** 10.1057/s41599-022-01356-5

**Published:** 2022-10-04

**Authors:** Maria Symeonaki, George Filandrianos, Giorgos Stamou

**Affiliations:** 1 School of Political Sciences, Department of Social Policy, Panteion University of Social and Political Sciences, Athens, Greece; 2 School of Electrical and Computer Engineering, National Technical University of Athens, Athens, Greece

**Keywords:** Social policy, Sociology, Science, technology and society

## Abstract

DGmap is an online interactive tool that visualises indicators drawn from large-scale European and international databases reflecting the use of information and communication technologies (ICT) amongst children and young individuals in Europe. A large number of indicators are estimated and visualised on an interactive map revealing convergences and divergences amongst European countries. Apart from its main feature, that of facilitating users to observe discrepancies between countries, the map offers the potentiality of downloading or customising country reports, information concerning the estimation of the indices and their values as spreadsheets, while covering a period from 2015 and onwards. DGmap also allows users to examine the evolution of each indicator through time for each country individually. Thus, the presented tool is a dynamic and constantly updated application that can serve as a major source of information for those interested in the use of digital technologies by children, adolescents, and young people in Europe.

## Introduction

Data and information visualisation is described as the process of representing data in a graphical and expressive manner, allowing the wider audience to look for or discover what is implied from large amounts of data in a well-organised and successful way. Data visualisation is specifically effective as a means of reading between the numbers and transforming raw data into eloquent graphical representations. Following Kirk (2019) data visualisation is defined as the representation and presentation of data to facilitate understanding. Representation refers to the act of attaining understanding from data by exhibiting it in a different visual form, such as a map, whereas presentation relates to interactivity choices, colours, or other displaying options one would have to consider.

The current paper presents an online interactive application, DGmap, that visualises the use of information and communication technologies (ICT) amongst children and young individuals in European countries, with data drawn from large-scale sample surveys, international and European. Apparently, there is a growing interest in the use of ICT or in the lack of access to ICT which may deepen the digital divide, especially in the post-COVID-19 era, where digital skills and digital capital play a key role in all educational processes (Cullinane and Montacute, 2020; Lai and Widmar, 2021; Lourenco and Tasimi, 2020; Frenette et al., 2020). The current study addresses issues of first- and second-level digital divide at a micro-level, i.e., not only access, but also skills, interest, and confidence in using ICT. The unprecedented pandemic may lead to new forms of social inequalities generating a new paradigm shift and global chain reactions concerning digital transformations. Possible factors explaining the digital gap are linked to socio-economic and demographic variables such as gender, disability, age, and socio-economic status (Ragnedda and Muschert, 2017; Senkbeil et al., 2019; Kuc-Czarnecka, 2020; Ayllón et al., 2021). The younger generation’s views on how their education is preparing them for the digital age in the context of the COVID-19 pandemic are also studied in Eickelmann et al. (2021).

Moving beyond the access and the discrepancies in ICT use for educational purposes, it is evident that ICT plays an essential role in the everyday life of families, children, and adolescents. The indices presented on DGmap cover specific thematic categories in relation to minors and young individuals: digital skills and digital deprivation, family and leisure, education, and online civic participation, with raw data drawn from (1) the European Union’s Statistics on Income and Living Conditions (EU-SILC), 2015–2020; (2) the OECD’s Programme for International Student Assessment (PISA), 2015 and 2018; (3) the European Social Survey (ESS), 2018; (4) the International Computer and Information Literacy Study (ICILS), 2018; (5) the 2nd ICT in Education Survey; and (6) the Trends in International Mathematics and Science Study (TIMMS), 2019. DGmap is a dynamic tool that is regularly updated with knowledge whenever new rounds from these databases are released, and new indicators are estimated. There are currently 52 indicators covering these broad thematic categories, such as the average time that students spend on the internet outside of school, the percentage of children that live in a household that cannot afford to have a computer and/or internet connection, the percentage of students that play collaborative online games every day, students’ interest or confidence concerning ICT, or the percentage of students that claim they have been cyberbullied when others shared embarrassing photos or hurtful material online. The main functionality of the map is to facilitate users to observe discrepancies between European countries, by visualising the indicators’ values on DGmap, with countries having increased values presented with more intense colour. Descriptive statistical measures are also provided, whereby users can detect the minimum and maximum values of the indicator and the countries to which they correspond, and the mean and standard deviation for all countries. Users can get information on countries by highlighting one or by downloading or customising country reports. Information concerning the estimation of the indices is also provided. DGmap also allows users to examine the evolution of each indicator through time for each country separately. Thus, the presented application is a dynamic and constantly updated instrument that can serve as a major source of information for those interested in the use of digital technologies by the younger generation.

The paper has been organised in the following way. Section “Methodology and technical specifications” presents the methodology and technical specifications that concern the development and operation of DGmap. Section “Description of DGmap's main functionalities” sketches DGmap’s main functionalities, whereas section “Information derived from large-scale databases” addresses issues concerning the international and European databases that were accessed and informs this work. The fifth section “Information and visualisation” gives an overview of the information that is visualised on DGmap, covering issues relating to indicator selection and the measurement of ICT use by children and young individuals. Finally, the concluding section “Discussion and future work” provides ideas for discussion, aspects, and suggestions for future work.

## Methodology and technical specifications

### Methodology

The work builds on earlier information visualisation paradigms in social research, such as the interactive map developed by the Erasmus University in Rotterdam, depicting the analysis of the total population, youth, migrants, and older individuals across 29 European countries, listing state-level data on two main aspects of labour market resilience: unemployment rate and at risk of poverty and social exclusion from 2007 to 2010 (http://www.inspires-research.eu/labourmarket). It also follows the logic behind Eurostat’s Statistical Atlas (https://ec.europa.eu/statistical-atlas/viewer/themes/), a map viewer where one can explore interactive maps for a range of different topics, such as population and census statistics, land use and land cover, etc. Eurostat also exhibits relevant information based on the survey on ICT use in households and by individuals in the form of Tables. Apparently, ICT changes occur at a fast pace, intensified by the COVID-19 pandemic, and by the time data are made available to researchers, and variables/indicators are identified and estimated, data becomes somehow outdated. There is therefore an apparent need for communicating relevant information at-a-glance the fastest possible. Few (2013) provides guidance for a useful information dashboards’ taxonomy, proposing three high-level categories: strategic, operational, and analytical. We opted for covering all three categories, and thus DGmap offers (i) an uncomplicated view of relevant indicators to enable rapid visual parsing to identify measures off target in cross-country comparisons, also offering qualitative information by highlighting worse and best-performing countries at-a-glance (strategic), (ii) longitudinal changes in indicators were available to identify trends (operational), and (iii) the possibility to identify relationships by downloading the estimated indicators in the form of spreadsheets (analytical). The suggested visualisation was proposed taking experts’ knowledge into consideration in relation to user/stakeholders needs plus the recommendations of an advisory board where DGmap was presented right before launch. Refinements to functionalities, design, and layout were made accordingly.

With ever-increasing options of ready-made systems for visualising data, a thorough investigation was needed, incorporating experts’ feedback in addition. There are basically three categories of ready-made systems for visualising data on a map, differentiated according to the complexity of the maps produced. The first visualisation method is often used in more straightforward tasks, such as data depicting a single indicator (Romero, 2019). It involves submitting properly structured files (e.g., CSV or XLSX files) to a page that subsequently manages the visualisation and returns a public URL for sharing. There is a plethora of online tools such as Google Maps, Heatmapper, Gunnmap, and Datawrapper. These tools assist non-specialists to upload data and create maps that communicate information in a straightforward way. However, they have limited potential in relation to the type, colour, or the map’s functionalities and environment, frequently preventing the addition of a menu, a second page, or even a logo. Besides, a drawback of ready-made systems in general, but which is more commonly observed in the first category, is that the produced maps become unavailable, as soon as the respective systems become inaccessible for whatever reason, with a typical example being that of OpenHeatMap. The second category includes applications such as Public Tableau, ArcGIS, and InstantAtlas that provide developers with additional customisation options. Several research papers (Akhtar et al., 2020; Pászto et al., 2020; Dong et al., 2020; Zhang, 2018) have used these techniques as a visualisation strategy for exhibiting data. These tools are usually more complex and require developers to investigate how to employ each option while constructing the map. They also provide advanced map functionalities, such as the ability to load multiple map data simultaneously or to select areas or countries to be displayed. However, it is often impossible to customise the map’s environment, and when allowed, there are limited options available. The final, more sophisticated category offers flexibility both in the map’s configuration and its context. The idea is to embed the map on a customised web page utilising a third-party online service. In this case, the page sends the data to an application that in return produces an instance of the map. The web page afterward needs to manipulate the response and display it appropriately in conjunction with its environment without restrictions. Apparently, this way of visualising data on a map requires a web developer, which is not compulsory in the first two categories. There are numerous such services, e.g., GeoCharts, Polymaps, D3.js, Mapbox, and Leaflet JS, and analogous studies in the literature that employ them (Worldometer, 2020; Zeng et al., 2019). As DGmap is a dynamic tool for visualising indicators with multiple functionalities, such as displaying user-customised charts, creating dynamic reports, downloading the respective data in the form of spreadsheets, or adding data for subsequent years, we selected the latter method using GeoCharts, ensuring the map’s unrestricted functionalities. GeoCharts is widely used and evaluated by various services in more demanding procedures. Other options were initially considered, such as Public Tableau, which was discarded due to limited preferences offered.

**Fig. 1:**
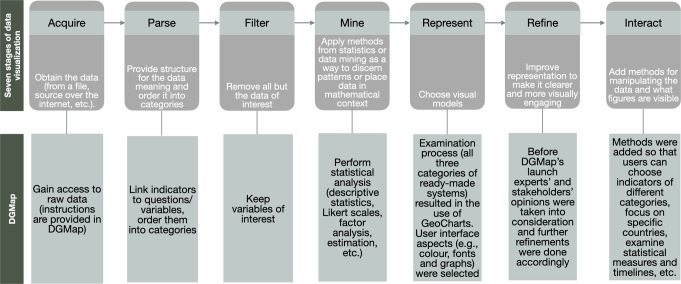
Stages of data visualisation and respective steps followed while developing DGMap. This figure depicts the stages summarised in Fry (2008) while visualising data, i.e., acquire, parse, filter, mine, represent, refine, and interact and the analogous steps followed while visualising data on DGmap.

Furthermore, data was visualised in line with the seven stages presented in Fry (2008), i.e., acquire, parse, filter, mine, represent, refine, and interact. Figure 1 depicts the stages summarised in Fry (2008) and the analogous steps followed while developing DGmap. Data representation (5th stage) was implemented on Fry’s (2008), Sedrakyan’s et al. (2019) and Schultz’s (2018) recommendations for visualising and communicating data in a clear, concise, and complete manner.

### Technical specifications

The application is divided into two main parts that communicate and operate effectively with each other as a single unit to assure DGmap’s effective performance: the frontend and the backend.

The user interacts directly with the part of the application called frontend, which concerns the appearance of the page, as well as the functionalities that run in the browser, such as button designs, the appearance of the map and autocomplete suggestions. DGmap’s user interface is developed using HTML5 (HyperText Markup Language), CSS3 (a language used to style an HTML document) and JavaScript. Specifically for the appearance and the embedment of responsive and sophisticated types of graphs, an open-source package called Bootstrap v.4.0 was used which offers sufficient choices for charts and multiple options for customisation. DGmap’s functional part on the client page uses a feature-rich JavaScript library, namely jQuery v.3.5.1. Like Bootstrap, jQuery is a library that contains a set of predefined complex JavaScript functions that makes HTML document traversal and manipulative, and event handling, animation, and Ajax simpler. Moreover, DGmap was developed using GeoCharts, a Google’s library, regularly employed to plot and highlight data per geographic area using varying levels of colour intensity and multiple levels of granularity for drilling down into countries. DGmap allows users to instantly shift from an overview of data to a more detailed and granular view within the same dataset by choosing a specific country, i.e., it enables users to study information from diverse perspectives by stepping up or down from one level of a predefined data hierarchy to another. For users to reload the map without having to reload the whole page, DGmap makes use of Javascript and jQuery. Finally, the charts were developed through Charts.js v.3.6.0, a package that includes a set of predefined functions for depicting graphs to users. Asynchronous Javascript and XML (AJAX) technology is used to allow DGmap to be updated asynchronously by exchanging data with the server in the background. This means that AJAX makes it possible to update parts of a web page, without reloading the whole page.

For the backend, we employ Python v.3.8.8, an open-source general-purpose programming language. To transform a local application into an online-micro-service Flask v.2.0.1 module is used together with Gunicorn v.20.1.0. In addition, DGmap makes use of a number of Python libraries to carry out this functionality on the server side, such as NumPy (Python’s numerical mathematics extension which adds support for large, multi-dimensional arrays and matrices, along with a large collection of high-level mathematical functions to operate on these arrays) and Matplotlib (a plotting library for Python and NumPy).

It is important to point out here that DGmap is freely open to users that do not need to complete any registration or login process. SLL encryption is used to guarantee privacy, authentication, and data reliability in the application’s platform. Finally, its operation has been tested and it performs unconcernedly on several browsers (Google Chrome, Safari, Mozilla Firefox and Microsoft Edge) using either MacOS, Microsoft, Android or a Linux Distribution.

## Description of DGmap’s main functionalities

**Fig. 2:**
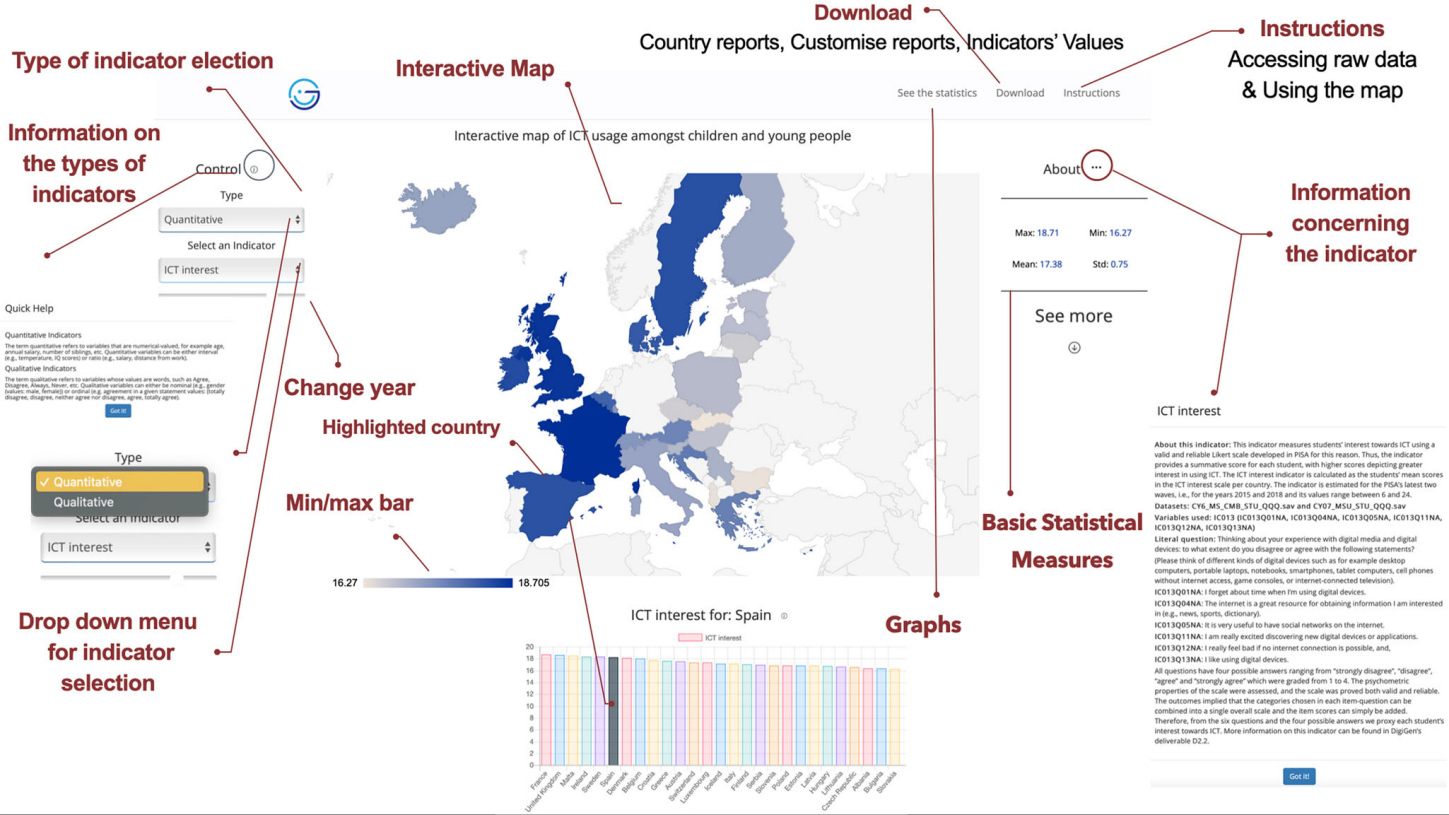
Main functionalities of DGmap. This figure expresses the frontend, and the main operations and functionalities of the application.

This Section describes in detail DGmap’s frontend, and the wide range of operations and functionalities it provides. Our main objective is to issue an online interactive map depicting information relating to ICT and the impact of technological transformation on the digital generation. However, other operations are available to help users establish an explicit picture on the thematic categories under consideration. Figure 2 expresses the main operations of the application.

Selecting an indicator automatically produces the respective map, where European countries are arranged according to the indicator’s values. The higher the indicator’s value, the greater the colour’s intensity. The user can hover over the countries and detect the indicator’s value for all countries. When the user clicks on a country it is highlighted, and the country’s position is designated on a min–max bar according to the respective value. This allows the user to interpret precisely where the country of interest rests in reference to the other countries.

Basic descriptive statistical measures are additionally exhibited, i.e., the minimum and maximum value, the mean value for all countries and the respective standard deviation. Hovering over the min and max values informs the user of the two countries that present the minimum and the maximum value. The user is transferred to the area where the graphs are produced (Fig. 2) by moving downwards or by clicking “See the Statistics”. The graphs relate to the indicator’s values for all countries, with the featured country highlighted in grey and either the evolution of the indicator’s values through time, when a quantitative indicator is selected or a bar chart of the categories of the qualitative indicator if the user opted for this choice.

More material in relation to the selected indicator is additionally accessible to assist users, i.e., social researchers, academics, policymakers and the wider public, in understanding what exactly is estimated via the selected indicator. Detailed information is therefore provided by clicking on the “About…” button and as a result, the required documentation for the indicator appears. The information provided for all indicators includes:

- the definition of the indicator that expresses exactly what the indicator measures,
- the linked dataset that was used to estimate the indicator,
- the exact variable(s) that are involved in the estimation of the indicator and the methodology followed for the indicators’ evaluation,
- the precise question that was used in the survey’s questionnaire and posed to respondents, and
- the values of the variable(s), i.e., the categories amongst which respondents can choose.

Guidance is available to users through two specific handbooks that give instructions for accessing raw data from the large-scale sample surveys that were used for the indicators’ evaluation, and for navigating through DGmap.

DGmap offers moreover the possibility to download country reports in pdf format, customised country reports, and the values for the selected indicator as spreadsheets in Excel.

## Information derived from large-scale databases

**Fig. 3:**
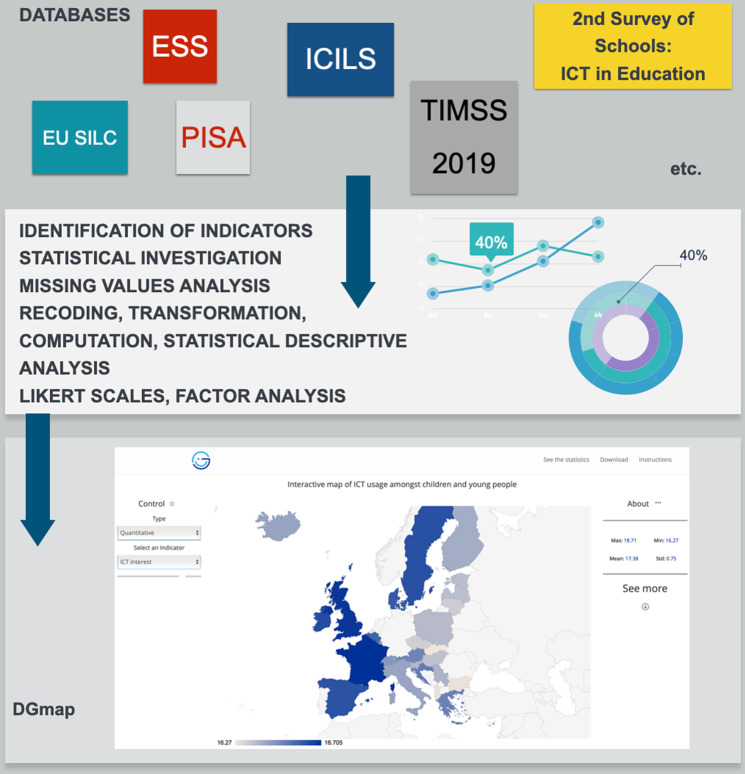
The interconnection between the databases, the indicators and DGmap. This figure presents the interconnection between the databases, such as the PISA and the ESS databases, the indicators, for example, average time spent on the Internet, and the application.

This section works through the specifics concerning the large-scale sample survey databases that were accessed to download the necessary raw data for the estimation of the indicators. A thorough list of available databases that allow both computing relative indicators and performing cross-national comparative analysis are presented in Ayllón et al. (2020). From the numerous databases presented in this report, DGmap makes use of the most relevant international and European databases, which are also available to researchers, academics, and policymakers free of charge for scientific purposes, and illustrate the multi-facet experience of ICT use by the younger generation in Europe. Figure 3 presents the interconnection between the databases, the indicators and DGmap. The databases are the main source of information that feeds DGmap, therefore it is of utmost importance to identify and use only reliable, large-scale, well-known sample surveys, which allow the computation of related child indicators in concern with ICT, but also cross-national comparative analysis. The main objective is to provide a comprehensive and elaborative picture of the multi-dimensional phenomenon of ICT use by the younger generation across Europe.

Up to this point, DGmap exhibits indicators that are derived from the following large-scale sample surveys.

### ICT in Education (2nd wave, students aged between 12 and 17)

The ICT in Education study (2nd Survey of Schools: ICT in Education, 2019) is linked to the Digital Education Action Plan (2021–2027), a renewed European Union policy initiative to foster the sustainable and effective adaptation of the education and training systems of EU member states to the digital age. The survey’s overarching goal is to provide data and evidence regarding the digitalisation in schools and more precisely by studying:

- the access to and use of digital technologies,
- the digital activities and digital confidence of teachers and students,
- ICT-related teacher professional development,
- the digital home environment of students, and
- the schools’ digital policies, strategies, and opinions.

Raw data included in all databases (schools’, parents’, and students’ questionnaires) is available to researchers for scientific purposes. Important information is provided concerning the access and use of digital technologies and devices, online activities and attitudes of teachers and students concerning ICT use, availability of devices, ICT and leisure activities, among others. Indicators derived from the ICT in education survey and exhibited on DGmap so far concern students who were aged between 12 and 17 years old at the time of the survey.

### PISA (2015 and 2018, 15-year-old students)

PISA is the OECD’s Programme for International Student Assessment (OECD, 2017; OECD, 2019) that has the main objective of measuring 15-year-old students’ reading and mathematics ability, science knowledge and abilities to meet real-life challenges. Raw data from the PISA database includes responses from students, school principals and parents, covers 79 OECD countries, and is freely available to researchers who would like to perform their own analysis. All variables included in DGmap so far are drawn from the students’ questionnaires, either the main questionnaire or the students’ ICT familiarity questionnaire. The student’s file is the source data file that incorporates students’ responses and scores, possible assessments for specific cognitive areas, such as ICT interest or ICT confidence and repetitive weights that should be used when analysing the data.

### The European Union Statistics on Income and Living Conditions Survey (2015–2020, children aged between 5 and 16)

The European Union Statistics on Income and Living Conditions Survey (EU-SILC) is one of Eurostat’s main studies which specifically intends to collect timely and comparable cross-national and longitudinal multidimensional raw data to monitor income, poverty, social exclusion, and the living conditions of Europeans, as part of the European framework for coordinating economic policies across the EU. Access to raw data is permitted only by agreement with Eurostat, and it is granted only for scientific purposes and upon a successful evaluation of a proposal to Eurostat’s microdata access team. The data includes interview survey data for adults aged 16 years and over and variables cover a wide range of topics such as primarily personal and household data, childcare, dwelling type, tenure status and housing conditions, housing costs and amenities, housing and non-housing related arrears, non-monetary household deprivation indicators, physical and social environment, household and personal level income, education, health and access to healthcare, and labour information. DGmap includes three indicators derived from data drawn from the EU-SILC, one of them is the percentage of digital-deprived children presented in Ayllón et al. (2021), where it was also suggested that digital deprivation should be considered as part of the definition of material deprivation used by the European Commission to monitor the progress of European societies.

### The European Social Survey (2018, young individuals aged between 15 and 24)

The European Social Survey (ESS) (European Social Survey, 2021) is an important source of information on the attitudes, beliefs, and behaviour patterns of individuals in more than thirty European countries. The ESS is a long-term research project, conducted every 2 years since 2002. Every 2 years, face-to-face interviews are conducted with newly selected, cross-sectional samples. The primary aims of the ESS are:

- to record stability and change in social structure, conditions, and attitudes in Europe and to interpret how Europe’s social, political, and moral fabric is changing,
- to achieve and spread higher standards of rigour in cross-national research in the social sciences, including, for example, questionnaire design and pre-testing, sampling, data collection, reduction of bias and the reliability of questions,
- to introduce soundly based indicators of national progress, based on citizens’ perceptions and judgements of key aspects of their societies,
- to undertake and facilitate the training of European social researchers in comparative quantitative measurement and analysis, and
- to improve the visibility and outreach of data on social change among academics, policymakers and the wider public.

DGmap makes use of raw data drawn from the latest round of the ESS to capture ICT habits and practises mainly concerning online civic participation.

### The International Computer Science and Literacy Study (2018, 13-year-old students)

The International Computer Science and Literacy Study (ICILS) (ICILS, 2018) brings together ample data on students’ ICT skills and use of computer science in their everyday life and habits, research, creativity, and communication. The study’s main interest is to interpret how well students in participating countries are prepared for study, work, and life in a digital era and to equip policymakers with an evaluation of educational programmes related to ICT. These concepts were put forward in the ICILS, 2018 assessment framework (Fraillon et al., 2019). The survey is carried out by the International Association for the Evaluation of Educational Achievement (IEA) in collaboration with the Australian Council for Educational Research (ACER-Australian Council for Educational Research). Raw data is freely available to researchers for scientific purposes.

### Trends in International Mathematics and Science Study (2019, 9- and 13-year-old students)

DGmap presents indicators that were estimated from data almanacks extracted from the latest cycle of Trends in International Mathematics and Science Study (TIMSS) (Mullis et al., 2020; Fishbein et al., 2021; TIMSS, 2019) conducted in 2019 at the fourth and eighth grades in 64 participating countries, supervised by IEA, the International Association for the Evaluation of Educational Achievement. The survey was inaugurated in 1995 and is carried out every 4 years, producing trends in mathematics and science achievement. In addition, TIMSS imparts data associated with policy recommendations concerning students’ contexts for learning mathematics and science based on students’, parents’, teachers’, and school principals’ questionnaires. TIMSS produces comparative timely, cross-national data on students’ achievement in relation to key home, school, and classroom variables. Up to this point, DGmap presents data from the eTIMMS students’ questionnaire.

## Information visualisation

**Fig. 4:**
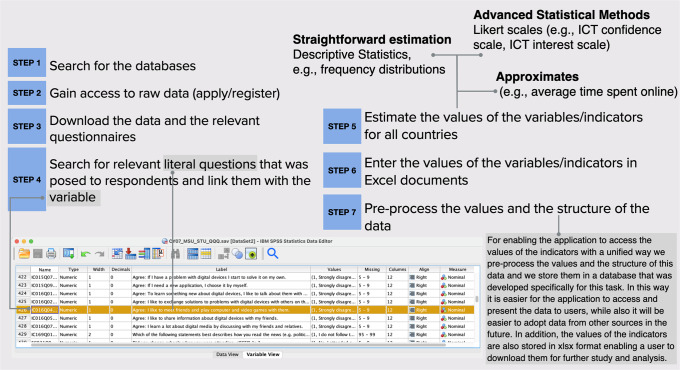
From the databases to DGmap. In this figure, an example of the process of getting from the databases to DGmap is provided with data downloaded from the PISA 2018 survey in SPSS.

This section provides detailed information concerning the indicators that are exhibited on DGmap. One of the main goals of this user-friendly visualisation tool is to introduce soundly based indicators for ICT use amongst children and young people in European countries, serving as an important source of integrated information that derives from relevant social sample surveys. Following the extensive literature review and outline of relevant existing data presented in Ayllón et al. (2020), DGmap contributes substantially to the development of social indicators reflecting online habits and practices of minors and young individuals. A long list of suitably robust indicators is currently available on DGmap tapping four broad domains of ICT use concerning this age category: digital skills and digital deprivation, education, family and leisure, and online civic participation. A number of indices related to common issues raised by minors and young individuals, but also by policymakers, parents and teachers, such as time spent on the Internet. Other indicators exploit domains that have been identified as missing from the common indicator set used in the literature. Specific examples include digital disengaged or deprived children (Ayllón et al., 2021). Figure 4 describes the sequential steps of the procedure followed for the estimation of the indicators, starting from the examination of the databases, and ending with the upload of the indicator’s values on DGmap. Apparently, the initial step would be to review the availability of relevant data sources. The identification of the database is followed by gaining access to raw data and downloading the data in an analysable form (e.g., in SPSS or SAS) together with the relevant questionnaires that were distributed during the survey. Searching for the exact questions that were posed and linking them with specific variables is the subsequent phase. In Fig. 4 an example of the database is provided with data downloaded from the PISA 2018 survey in SPSS, i.e., CY07_MSU_STU_QQQ.sav. The literal question that was posed can also be seen “How often do you use digital devices for playing collaborative online games outside of school”, with values: Never or hardly ever, Once or twice a month, Once or twice a week, Almost every day, Every day. The name of the variable is IC016Q04NA. The estimation of the indicators’ values is in most cases a quite straightforward descriptive statistical procedure, nevertheless, it sometimes requires more advanced statistical methods like Likert scaling or transforming the data to reach an estimation or approximation of the indicator’s values. All data were weighted according to the instructions of the source databases, e.g., anweight (analysis weight) was used to weight data drawn from the ESS, correcting for differential selection probabilities within each country as specified by sample design, W_FSTUWT (final trimmed nonresponse adjusted student weight) was used for cases drawn from the PISA survey, etc. Having estimated the values lead us to the next step of importing the data into spreadsheet documents and pre-processing the values and the structure of the data. This is necessary for enabling the application to access the values of the indicators in a unified way, storing the values in a database that was developed especially for this task. It is then simpler for DGmap to access and display the indicators to users, but also easier to adopt data from other sources in the future. The indicators’ values are also stored as Excel files to enable users to download them for further research.

However, this is not just about putting a number against ICT use by children. DGmap fills the need for comparable indicators over time and across national borders in this particular matter. For each indicator statistical, descriptive, structural, and reference metadata is provided to bring forth information about the indicators. This will assist users in understanding what was actually measured through the selected indicator, which age category it refers to, what database was used, what was the literal question that was posed to minors, and which are the variables that were analysed in case one would want to replicate the estimation, together with their values. Instructions are also provided for accessing raw data from the sample surveys that were used to estimate the indicators and for using the interactive map.

The indicators per domain of interest are presented in Tables 1–4. Knowing that some indicators can be related to more than one category, we emphasise here that they are placed in Tables 1–4 based on the highest relevance to a domain, but this is not reflected in the way the indicators are depicted on DGmap exactly for this reason.

**Table 1:** Digital capital and digital deprivation indicators.

Indicator	Description
Digital deprivation	The percentage of children aged 5–16, that live in a household that cannot afford to have a computer and/or internet connection.
Computer access	The percentage of children aged 5–16, that live in a household that cannot afford to have a computer.
Internet access	The percentage of children aged 5–16, that live in a household that cannot afford internet connection.
ICT interest	15-years-old students’ interest towards ICT is estimated using a valid and reliable Likert scale as a summative score, with higher scores depicting greater interest in using ICT. The indicator’s values per country are calculated as the students’ mean scores in the ICT interest scale.
ICT confidence	15-years-old students’ confidence in using ICT is estimated using a valid and reliable Likert scale as a summative score, with higher scores depicting greater confidence in using ICT. The indicator’s values per country are calculated as the students’ mean scores in the ICT confidence scale.
Digital disengaged students	The percentage of 15-years-old students that are digitally disengaged and lack interest towards ICT.
Digitally uncomfortable students	The percentage of 15-years-old students that lack confidence towards ICT.
Sense of time and ICT	The percentage of 15-years-old students that never forget about time when they use digital devices.
No excitement for digital devices and applications	The percentage of 15-year-old students that are not at all excited about discovering new digital devices or applications.
Dislike using digital devices	The percentage of 15-year-old students that totally dislike digital devices.
Uncomfortable with less familiar digital devices	The percentage of 15-year-old students that are totally uncomfortable using digital devices that they are less familiar with.
Uncomfortable with digital devices at home	The percentage of 15-year-old students that are totally uncomfortable using their digital devices at home.
Internet use	This indicator measures Internet use of young individuals (percentage frequency distribution to the categories: never, only occasionally, a few times a week, most days, every day).

**Table 2:** Technological transformation in families and leisure.

Indicator	Description
Average time spent on the Internet outside of school (per week)	Average time (in hours) that 15-year-old students spend on the Internet outside of school per week.
Average time spent on the Internet (per week)	Average time (in hours) that 15-year-old students spend on the Internet per week (outside of school and at school).
Age of first access to the Internet	The percentage frequency distribution of the age that 15-year-old students first accessed the Internet: 3 years old or younger; 4–6 years old; 7–9 years old; 10–13 years old; older than 13; I have never accessed the Internet.
Age of first use of digital devices	The percentage frequency distribution of the age that 15-year-old students first used a digital device: 3 years old or younger; 4–6 years old; 7–9 years old; 10–13 years old; older than 13; I have never used a digital device until today.
Internet connection at home	The percentage frequency distribution of the availability of Internet connection at home for 15-year-old students (Yes, and I use it; Yes, but I don’t use it; No).
Chatting online outside of school	The percentage frequency distribution of the use that 15-year-old students make of digital devices outside of school for chatting online outside of school (Never or hardly ever; Once or twice a month; Once or twice a week; Almost every day; Every day).
Browsing the Internet for fun	The percentage frequency distribution of the use that 15-year-old students make of digital devices outside of school for browsing the Internet for fun (Never or hardly ever; Once or twice a month; Once or twice a week; Almost every day; Every day).
Downloading music, films, games or software	The percentage frequency distribution of the use that 15-year-old students make of digital devices outside of school for downloading music, films, games or software from the Internet (Never or hardly ever; Once or twice a month; Once or twice a week; Almost every day; Every day).
Downloading new apps on a mobile device	The percentage frequency distribution of the use that 15-year-old students make of digital devices outside of school for downloading new apps on a mobile device (Never or hardly ever; Once or twice a month; Once or twice a week; Almost every day; Every day).
Participation in social networks	The percentage frequency distribution of the use that 15-year-old students make of digital devices outside of school for participating in social networks, such as Facebook, etc (Never or hardly ever; Once or twice a month; Once or twice a week; Almost every day; Every day).
Playing collaborative online games	The percentage frequency distribution of the use that 15-year-old students make of digital devices outside of school for playing collaborative online games (Never or hardly ever; Once or twice a month; Once or twice a week; Almost every day; Every day).
Playing one-player games	The percentage frequency distribution of the use that 15-year-old students make of digital devices outside of school for playing one-player games (Never or hardly ever; Once or twice a month; Once or twice a week; Almost every day; Every day).
Playing online games via social networks	The percentage frequency distribution of the use that 15-year-old students make of digital devices outside of school for playing online games via social networks (Never or hardly ever; Once or twice a month; Once or twice a week; Almost every day; Every day).
Sharing own created content	The percentage frequency distribution of the use that 15-year-old students make of digital devices outside of school for uploading their own created contents for sharing (Never or hardly ever; Once or twice a month; Once or twice a week; Almost every day; Every day).
Like to meet friends and play computer and video games	The percentage frequency distribution of the agreement of 15-years-old students to a given statement that was posed concerning whether they like or dislike meeting friends and playing computer and video games with them (Strongly agree; Agree; Disagree; Strongly disagree).
ICT use for communicating with friends, family, or other people using instant messaging, voice, or video chat	The indicator provides the percentage frequency distribution of ICT use for communicating with friends, family, or other people using instant messaging, voice, or video chat (Never; Less than once a month; At least once a month but not every week; At least once a week but not every day; Every day).

**Table 3:** ICT in education.

Indicator	Description
Available computer at home for doing schoolwork	The percentage frequency distribution of the availability of a computer at home for 15-year-old students to use for schoolwork (Yes; No).
Use digital devices at school for doing homework on a school computer	The percentage frequency distribution of the use that 15-year-old students make of a school computer for doing homework (Never or hardly ever; Once or twice a month; Once or twice a week; Almost every day; Every day).
Use digital devices at school for downloading, uploading, or browsing material from the school’s website	The percentage frequency distribution of the use that 15-year-old students make of digital devices at school for downloading, uploading, or browsing material from the school’s website (Never or hardly ever; Once or twice a month; Once or twice a week; Almost every day; Every day).
Posting work on the school’s website	The percentage frequency distribution of the use that 15-year-old students make of digital devices at school for posting their work on the school’s website (Never or hardly ever; Once or twice a month; Once or twice a week; Almost every day; Every day).
Use of learning apps or learning websites	The percentage frequency distribution of the use that 15-year-old students make of digital devices at school for learning apps or learning websites (Never or hardly ever; Once or twice a month; Once or twice a week; Almost every day; Every day).
Use of school computers for group work and communication with other students	The percentage frequency distribution of the use that 15-year-old students make of digital devices at school for group work and communication with other students (Never or hardly ever; Once or twice a month; Once or twice a week; Almost every day; Every day).
Use of digital devices outside of school for browsing the Internet for schoolwork	The percentage frequency distribution of the use that 15-year-old students make of digital devices at school for browsing the Internet for schoolwork (e.g., for preparing an essay or presentation) (Never or hardly ever; Once or twice a month; Once or twice a week; Almost every day; Every day).
Browsing the Internet outside of school to follow up lessons	The percentage frequency distribution of the use that 15-year-old students make of digital devices outside of school for browsing the Internet to follow up lessons, e.g., for finding explanations (Never or hardly ever; Once or twice a month; Once or twice a week; Almost every day; Every day).
Use digital devices outside of school for doing homework on a computer	The percentage frequency distribution of the use that 15-year-old students make of digital devices outside of school for doing homework on a computer (Never or hardly ever; Once or twice a month; Once or twice a week; Almost every day; Every day).
Use digital devices outside of school for communication with other students about schoolwork via email	The percentage frequency distribution of the use that 15-year-old students make of digital devices outside of school for communication with other students about schoolwork via email (Never or hardly ever; Once or twice a month; Once or twice a week; Almost every day; Every day).
ICT and the future	This indicator reflects the adolescents’ attitude towards ICT impact on their future constructed via a Likert scale. The values of this indicator range between 4 and 16, with higher values associated with adolescents that think higher of ICT’s impact on their future. The relevant questions are: It’s really worth using a computer because it will help me in the future; I use a computer to learn as it will help me in the work that I want to do later on; I learn things using computers that will help me to get a job; Learning with computer is important for me because I need it for what I want to study later on.
Impact of ICT use during lessons	This indicator reflects the adolescents’ attitude towards ICT impact, constructed via a Likert scale. The values of this indicator range between 7 and 28, with higher values associated with adolescents that think higher of ICT’s impact on education. The literal questions ask whether they consider that using ICT during lessons has a positive impact on the following: You concentrate more on what you’re learning; You try harder in what you are learning.; You feel more independent in your learning; You understand more easily what you’re learning; You remember more easily what you’ve learnt; ICT enables you to work better with other students on tasks; ICT improves the atmosphere in class (students are more engaged, there is less disruption).
Harmful messages online	The frequency that 8th-grade students report that they have been sent nasty or hurtful messages online during the school year of the survey (At least once a week; Once or twice a month; A few times a year; Never).
Shared hurtful things online	The frequency that students report others share hurtful things about them online during the school year of the survey (At least once a week; Once or twice a month; A few times a year; Never).
Shared hurtful photos online	The frequency that students report that others share embarrassing photos of them online during the school year of the survey (At least once a week; Once or twice a month; A few times a year; Never).

Indicators concerning online civic participation are fewer in number, however, there is some information available. Indicators estimated with data drawn from the ESS refer to a broader age category (15–24) due to sample size restrictions.

**Table 4:** ICT and transformation of civic participation (digital citizens).

Indicator	Description
Participation in online group discussions or forums	The percentage frequency distribution of the participation of 15-year-old students in online group discussions or forums (I don’t know what that is; Never or almost never; Several times a month; Several times a week; Several times a day).
Reading online news	The indicator provides the percentage frequency distribution of 15-year-old students and their activity of reading online news (I don’t know what that is; Never or almost never; Several times a month; Several times a week; Several times a day).
Reading news on the Internet outside school	The indicator provides the percentage frequency distribution of 15-year-old students being informed about the news through the Internet (Never or hardly ever; Once or twice a month; Once or twice a week; Almost every day; Every day).
Searching information online to learn about a particular topic	The indicator provides the percentage frequency distribution of 15-year-old students and their searching for information online to learn about a particular topic (I don’t know what it is; Never or hardly ever; Once or twice a month; Once or twice a week; Almost every day; Every day).
Interest in politics	This indicator measures the interest of young individuals in politics (percentage frequency distribution).
Confidence in participating in politics	The percentage frequency distribution of young individuals and their confidence to participate in politics (Not at all confident; A little confident; Quite confident; Very confident; Completely confident).
Signing petitions	This indicator provides information on whether young individuals have signed a petition during the last 12 months (in the form of the percentage frequency distribution: Yes; No).
Online posting or sharing concerning politics	The percentage frequency distribution of young individuals posting or sharing anything about politics online during the last 12 months (Yes; No).

## Discussion and future work

**Fig. 5:**
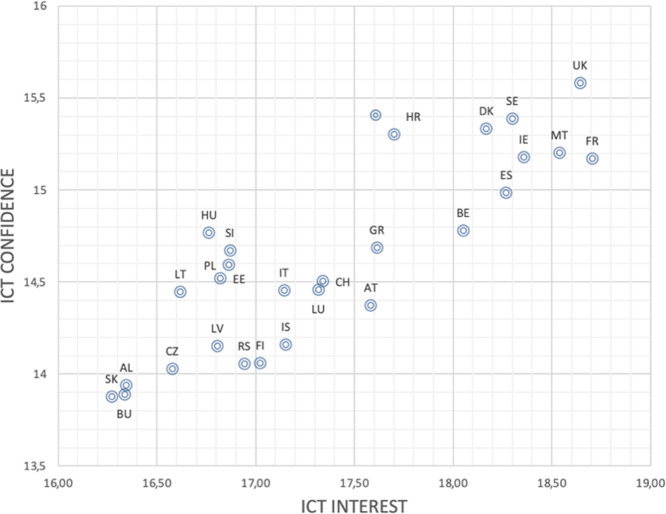
ICT interest and ICT confidence, PISA, 2018. This figure depicts an example of downloading the respective values of ICT interest and ICT confidence from DGMap and producing the respective scatterplot to observe relationships.

The main impetus behind the development of DGmap is the exhortation to systematically record, as well as suggest new indicators to measure ICT use and the children’s readiness to browse into a digital future. What has led to a renewed focus on this matter turning it recently into an urgent policy concern was the COVID-19 pandemic and its aftermath, although the changing nature and use of the Internet have long been identified as a mirror of social inequalities and a determinant of transformations in social stratification (Witte and Mannon, 2010). Quantitative indicators deriving from reliable large-scale sample surveys might provide a nuanced picture of children's digital habits, skills, confidence, or deprivation in Europe giving well-founded estimates of their digital life, whether subjective (if they feel confident for example in using ICT) or objective (the existence of Internet connection or a computer they can use at home). DGmap provides a user-friendly way that handles the multi-dimensional nature of this matter and simultaneously presents relative country differences to a previous time period or in terms of its position on a min–max European scale. All these indicators are needed in order to understand quantitative relationships and to gradually build up models, if, for example, one would like to identify the socio-economic and demographic characteristics that define children with a special interest or confidence in ICT or no interest at all. Moreover, an important feature of DGmap is the metadata information it provides as we need in this kind of an endeavour to be clear about what is being measured and how. We note here that sophisticated statistical analyses, such as confirmatory factor analysis, have already been performed in numerous cases, to prove unidimentionality of Likert scales for instance, in order to assist researchers with limited statistical experience in estimating and using advanced indicators, such as ICT interest and ICT confidence. Correlation between quantitative variables can be explored by downloading the respective indicators values as spreadsheets and further exploring their relationship. Figure 5 for example depicts the scatterplot of ICT interest and ICT confidence produced by downloading the respective values in Excel and further developing the graph to observe relationships.

Looking at DGmap one can conclude that ICT affects children’s everyday life in many ways, whether in school, at home or on the move. The use of ICT is widespread, however, looking at access to ICT at home for children aged between 5 and 16, we notice that approximately one out of 20 households in European countries cannot afford to have a computer. Between 2015 and 2019 the proportion of households in Europe that cannot provide a computer at home dropped slightly from 4.89% to 4.12%. Respective percentages of children of the same age that live in households that cannot afford Internet connection decreased from 5% to 2.53% between the same years. A combination of the two indicators that explores the percentage of children that live in digitally deprived households (Ayllón et al., 2021), with no computer and/or internet connection reveals that the share of the respective households dropped from 6.74% to 4% between 2015 and 2020 at the European level. On the other hand, in 2020 Romania recorded the highest rate of digitally deprived children among children aged 5–16 which was more than 23%, while in Estonia and Norway the respective percentage did not exceed 1% (0.57% and 0.63%, respectively).

The mean weekly Internet use outside of school for 15-year-old students in 29 European countries in 2018 is about 27 h a week, whereas the average time spent on the Internet at school (hours per week) is almost equal to 8 h a week. The highest weekly Internet use among adolescents at the age of 15, outside of school was recorded in Sweden (32.4), the UK (30.36) and Malta (29.60). By contrast, the lowest weekly hours of young people making daily use of the Internet outside school were recorded in Georgia (24.22), Slovenia (23.37) and Albania (20.14). Denmark and Sweden have the highest weekly hours for Internet use at school (17.80 and 14.01, respectively), while Ireland and Malta the lowest (4.16 and 3.98). Moreover, adolescents in Slovakia exhibit the lowest interest and confidence in ICT and on the other end we find French students that show the highest interest and adolescents in the UK that feel the most confident.

Similar conclusions can be drawn for other indicators included in the map that aid the mapping of the digital habits of European children and adolescents. DGmap’s ambition is to serve as a leading source of updated information as new waves from relevant databases are released, with the eventual set of indicators being a comprehensive collection in one place and a concentration of novel supplements to the existing stock of indicators. In a later stage, new data and indicators will be incorporated, from databases such as the ESS (2020), the PISA (2022), the ICILS (2023), the TIMMS (2023), which will capture post-COVID-19 difficulties and the socio-economic gap in children's digital competences.

## Data Availability

The estimated values of all indicators are available for downloading in the form of spreadsheets, i.e., the indicators’ values are stored as separate Excel files to enable users to download them for further research (Download → Download Excel) (https://apps.islab.ntua.gr/dgmap/). Moreover, instructions for accessing raw data are also provided (https://apps.islab.ntua.gr/dgmap/download-instructions/Instructions_Access_Data.pdf).
